# mTOR‐autophagy promotes pulmonary senescence through IMP1 in chronic toxicity of methamphetamine

**DOI:** 10.1111/jcmm.15841

**Published:** 2020-09-11

**Authors:** Mei‐Jia Zhu, Bing‐Yang Liu, Lin Shi, Xin Wang, Yun Wang

**Affiliations:** ^1^ Department of Clinical Pharmacology School of Pharmacy China Medical University Shenyang China; ^2^ Department of Endocrinology Shengjing Hospital of China Medical University Shenyang China

**Keywords:** autophagy, IMP1, methamphetamine, mTOR, pulmonary, senescence

## Abstract

It is growingly concerned about methamphetamine (MA)‐induced lung toxicity. IMP1 is identified as a key molecule for cell life processes, but the role of IMP1 in MA‐induced senescence remains unclear. The purpose of this study was to investigate whether chronic exposure to MA can cause autophagy and senescence of the lungs, whether there are interactions between Mammalian target of rapamycin (mTOR) and IMP1 and whether IMP1 is involved in pulmonary senescence promoted by mTOR‐autophagy. The rats were randomly divided into control group and MA group, following by H&E staining, immunohistochemistry staining and Western blot. The alveolar epithelial cells were proceeded by ß‐galactosidase staining, cell cycle detection, transfection and co**‐**immunoprecipitation. Long‐term exposure to MA led to the thickening of alveolar septum and more compact lungs. MA promoted the conversion of LC3‐I to LC3‐II and inhibited the activation of mTOR to induce autophagy. Bioinformatics and co**‐**immunoprecipitation results presented the interactions between IMP1 and mTOR. MA induced cell senescence by decreasing IMP1, up‐regulating p21 and p53, arresting cell cycle and increasing SA‐β‐gal. Overexpression of IMP1 reduced p21 and SA‐β‐gal to inhibit the senescence of alveolar epithelial cells. These results demonstrated that mTOR‐autophagy promotes pulmonary senescence through IMP1 in chronic toxicity of methamphetamine.

## 1 INTRODUCTION

Methamphetamine (MA) abuse has been a growing global health problem.^1^, ^2^ The highest absorption and distribution of MA in the lungs are extremely vulnerable to pulmonary infections, pulmonary hypertension and oedema.^3^ Lung toxicity by long‐term exposure to MA is very harmful to health.^4^ Alveolar epithelial cells in defence against infections and toxins are the key barriers to maintain the pulmonary homeostasis.^5^, ^6^ Therefore, alveolar epithelial injury is an important pathological change of MA‐induced lung toxicity.

Autophagy refers to a process by which cellular components are degraded by lysosomes or vacuoles.^7^ Mammalian target of rapamycin (mTOR) is universally acknowledged as an inhibitor of autophagy, so that autophagy will occur after mTOR is inactivated.^8^ Our previous studies found that MA‐induced inactivation of mTOR promoted autophagy‐mediated alveolar epithelial injury.^9^

Senescence is the complex process of deterioration that occurs over the lifetime of an organism, resulting in progressive functional decline and eventual death.^10^ Cellular senescence is a phenomenon that the cells lose the ability of self‐proliferation.^11^ Studies have shown that drug abuse, such as MA commonly used in HIV patients, can cause cell senescence and contributes to age‐related diseases in nervous system,^2^, ^12^ but there are few studies on the effect of MA‐induced senescence in the lungs. Although there are studies about the relationship between autophagy and senescence, their regulatory mechanism has been controversial. On the one hand, it is believed that autophagy is inhibited in ageing organisms, leading to the accumulation of harmful substances and damaged organelles.^13^, ^14^, ^15^ On the other hand, it is reported that autophagy is positively regulating senescence, that is, the senescence process is accompanied by autophagy.^16^, ^17^ However, it has been unknown about the relationship between autophagy and senescence in MA‐induced lung toxicity.

Insulin‐like growth factor 2 messenger RNA‐binding protein 1 (IGF2BP1, also known as IMP1) is a highly conserved RNA‐binding protein and mainly expressed in embryonic stage but less expressed during adulthood and expressed in malignant transformation.^18^, ^19^, ^20^ IMP1 can also stabilize c‐Myc mRNA and prevent its binding with specific sequences to suppress its degradation to increase the protein level of c‐Myc and promote cell proliferation accordingly.^21^, ^22^ Recovery of IMP1 expression in malignant cells not only promotes tumour progression, but also participates in the establishment and maintenance of tumour cell levels and increase the invasiveness of cancer.^19^, ^23^ However, IMP1 in breast cancer cells was significantly lower than that in colorectal cancer cells, and IMP1 had a positive role in inhibiting the growth and metastasis of breast cancer.^24^

IMP1 is related to PI3K, mTOR and MAPKs pathways through protein‐protein interactions.^21^, ^23^, ^25^ It is generally acknowledged that mTOR acts as an autophagy inhibitor to regulate cellular autophagy.^26^, ^27^, ^28^, ^29^ Studies have shown that mTOR could catalyse the phosphorylation of IMP1 at Ser181 site and that IMP1 could bind to mRNA of mTOR.^30^ Meanwhile, IMP1 deletion increases autophagy in Crohn's disease and ulcerative colitis.^31^ Therefore, we make a hypothesis that there may be a protein‐protein interaction between mTOR and IMP1. It is reported that IMP1 down‐regulation can inhibit cell proliferation and induce cellular senescence.^32^ Some studies have shown that the expression of p21, an indicator of senescence, is increased after IMP1 is knocked down in hepatocellular carcinoma, suggesting that IMP1 could regulate p21 expression to take a part in cellular senescence.^33^, ^34^ And it has also been reported that knocking down of IMP1 in hepatocellular carcinoma can result in G1 phase cell cycle arrest.^34^ Based on the above, the current study is aimed to investigate whether chronic exposure to MA can cause pulmonary autophagy and senescence, whether there are interactions between mTOR and IMP1 and whether IMP1 is involved in pulmonary senescence promoted by mTOR‐autophagy in chronic toxicity of MA.

## 2 MATERIALS AND METHODS

### 2.1 Animal models

Twenty male Wistar rats were obtained from the Animal Resource Center of China Medical University. They were divided into control group and MA group randomly. MA (China Criminal Police University, China) was injected intraperitoneally with the dosage of 10 mg/kg in the 1st week, and then, the daily dose was increased by 1 mg/kg per week, until the daily dose was increased to 15 mg/kg in the 6th week.^35^ The control group was given intraperitoneal injection with the equivalent of 0.9% physiological saline. The rats were group housed in air‐conditioned (the temperature is 18‐22°C and the humidity is 50%‐70%) rooms with a 12‐hour light cycle and allowed free access to food and water. All the rats were weighted every day. The percentage of weight gain in each group was calculated every week as the following Equation 1. After 6 weeks, the right ventricular index (RVI) was also calculated using Equation 2 to evaluate the remodelling of the right heart resulting from chronic pulmonary dysfunction. All experimental procedures involving animals were conducted following the guidelines of the Guide for the Care and Use of Laboratory Animals of the National Institutes of Health (NIH), with the approval of the Institutional Animal Care and Use Committee of China Medical University (IACUC Issue No. CMU2019215).

(1) $$\text{The}\;\text{percentage}\;\text{of}\;\text{weight}\;\text{growth}\;=\;\frac{\text{average}\;\text{of}\;\text{the}\;\text{weekly}\;\text{weight}\;‐\;\text{average}\;\text{of}\;\text{the}\;\text{initial}\;\text{weight}}{\text{average}\;\text{of}\;\text{the}\;\text{initial}\;\text{weight}}\;\times \;100\%$$

(2) $$\text{The}\;\text{right}\;\text{ventricular}\;\text{index}\;=\;\frac{\text{right}\;\text{ventricular}\;\text{weight}}{\text{right}\;\text{ventricular}\;\text{weight}\;+\;\text{left}\;\text{ventricular}\;\text{weight}}\;\times \;100\%$$

### 2.2 Cell culture and drug treatment

Alveolar epithelial cell lines A549 were obtained from the Experimental Center of China Medical University. Cells were cultured in RPMI‐1640 medium which contains 10% foetal bovine serum and 1% penicillin/streptomycin at 37°C under the condition of air:CO_2_ = 95:5. Cells were treated by MA (China Criminal Police University) with the dosage of 0.1, 0.5, 1 and 5 mmol/L for 6, 12 and 24 hours.^9^, ^36^

### 2.3 Transfection

The overexpression plasmids of IMP1 were designed and obtained from the GeneChem Group, Ltd. Cells were inoculated and transfected with plasmids when 70% of the culture flask were covered. The protocol is referred to the Lipofectamine™ 3000 Transfection Reagent (Thermo Fisher Scientific). The DNA and Lipo3000 were diluted with serum‐free medium, respectively, and then mixed together at a 1:1 ratio. After incubation 10‐15 minutes at room temperature, the mixture was added into normal cell medium. The cells were cultured at 37°C for 2‐4 days. Drugs were administrated at the time of highest transfection efficiency. Drug‐treated cells were analysed by Western blot and senescence associate ß‐galactosidase (SA‐ß‐gal) staining (Nikon MODEL ECLIPSE Ts2‐FL).

### 2.4 Cell cycle detection

The protocol is referred to the instruction of the cell cycle testing kit (KeyGEN Biotech). PBS or MA‐treated alveolar epithelial cells were collected, washed and then centrifuged at 2000 rpm for 5 minutes. The precipitated cells were fixed with 70% cold ethanol at 4°C overnight, following by washing and dyeing. Each sample was dyed with 500 μL PI/RNase A (9:1) dyeing solution for 30‐60 minutes in a dark room, then detected and analysed by flow cytometry (Bio‐Rad).

### 2.5 Western blot analysis

After sodium dodecyl sulphate‐polyacrylamide gel electrophoresis (SDS‐PAGE), proteins were transferred onto a PVDF membrane. The PVDF membrane was blocked for 2 hours and then incubated with corresponding primary antibodies (Table 1) at 4°C overnight. And they were incubated with secondary antibodies **(**HRP‐conjugated AffiniPure Goat Anti‐Rabbit IgG (H + L), 1:1000/2000/3000; Proteintech, SA00001‐2; HRP‐conjugated AffiniPure Goat Anti‐Mouse IgG (H + L), 1:6000/1:8000; Proteintech, SA00001‐1) for 2 hours in room temperature in the next day. After washing with PBS for five times, the membranes were detected by enhanced chemiluminescence (Proteintech).

**TABLE 1:** The primary antibodies of correlative proteins

Primary antibody	Dilution	Company	Catalogue
LC3	1:1000	Cell Signaling Technology, USA	#3868
mTOR	1:1000	Proteintech, USA	20657‐1‐AP
p‐ mTOR	1:500	Bioworld Technology, USA	BS4706
IMP1	1:1000/1:500(IHC)	Proteintech, USA	22803‐1‐AP
p21	1:1000	Proteintech, USA	10355‐1‐AP
p21	1:200/1:500(IHC)	Boster, China	BA0272
p53	1:1000	Proteintech, USA	10442‐1‐AP
β‐actin	1:10 000	Proteintech, USA	60008‐1‐lg

### 2.6 H&E and Immunohistochemistry (IHC) staining

The lung was infused with 0.9% physiological saline and fixed slowly with the 4% paraformaldehyde solution through airway. The opening of the airway was upward. After the lower part of the lung was 3/4 inflated, the trachea was tighten, and then, the lung was immersed in the fixed solution for more than 24 hours for paraffin embedding. The lung was sliced into 4‐µm sections for morphological measurements. H&E staining was to evaluate the thickness of alveolar septum and the number of alveolar sacs to illustrate lung injury (three fields of view randomly selected were analysed in each section; magnification, ×200, ×400).

Tissue paraffin sections were performed with the Ultrasensitive TM SP (Rabbit) IHC Kit (MXB Biotechnologies). The slides were incubated with IMP1 and p21 antibody, respectively, at 4°C overnight, incubated with secondary antibody for 10 minutes and then reacted with conjugated streptavidin‐peroxidase for 10 minutes at room temperature. The sections were observed under light microscope (Nikon MODEL ECLIPSE Ts2‐FL). The expression of IMP1 and p21 was calculated with optical density value.

### 2.7 Senescence‐associated ß‐galactosidase staining

The protocol was referred to the instruction of the senescence‐associated ß‐galactosidase staining kit (Beyotime Biotechnology). Briefly, a suitable amount of SA‐ß‐gal staining stationary solution was added into the cells treated by PBS or MA and then fixed at room temperature for 15 minutes. After washing with PBS for three times, a sufficient amount of pre‐prepared dyeing working liquid was added and incubated overnight in the incubator without CO_2_ at 37°C. The working fluid was removed in the next day, washed with PBS and observed under a microscope (Nikon, ECLIPSE TE2000‐U). The blue‐stained cells were identified as senescent cells and were counted in ten fields of view randomly selected in each section to determine the percentage of ß‐galactosidase‐positive cells (magnification, ×200).

### 2.8 Bioinformatics prediction

The structures of IMP1 and mTOR are from RCSB PDB data bank. MOE software is used to dock model structure of mTOR with IMP1. The binding sites between them are analysed by MOE software (CloudScientific Technology Co., Ltd).

### 2.9 Co‐Immunoprecipitation

Cells in two 10 cm × 10 cm plates were prepared. Beads (Protein A/G PLUS‐Agarose; Santa Cruz Biotechnology, sc‐2003) were washed with lysis buffer by rotating and then were added the equal volume of lysis buffer. The lysate was retained at 40 μL each as input. Beads were added into the lysate. The lysate was rotated with beads at 4°C for 1 hour followed by centrifugation at 1500 rpm for 3 minutes. Supernatant was retained in the new EP tubes. Two microgram IgG (Rabbit Immunoglobulin G; Dingguo Changsheng Biotechnology) and 2 μg target protein antibody mTOR were added to each tube, respectively, and rotated at 4°C overnight. In the next day, it was followed by centrifugation and discarding supernatant. Then, the beads were washed for three times with lysis buffer. The samples was added by appropriate 2× loading buffer and boiled at 100°C for 5 minutes for Western blot analysis.

### 2.10 Statistical analysis

All the data are expressed as mean ± SD. Statistical analysis was carried out by GraphPad Prism 5.0 (GraphPad Software, Inc). Non‐parametric *t* test (Mann‐Whitney test) and two‐way analysis of variance (ANOVA) were used for statistical comparisons. ANOVA were followed by Bonferroni post‐test. *P* < .05 was considered to have statistical significance.

## 3 RESULTS

### 3.1 Chronic exposure to MA‐induced pulmonary injury in rats

**FIGURE 1:**
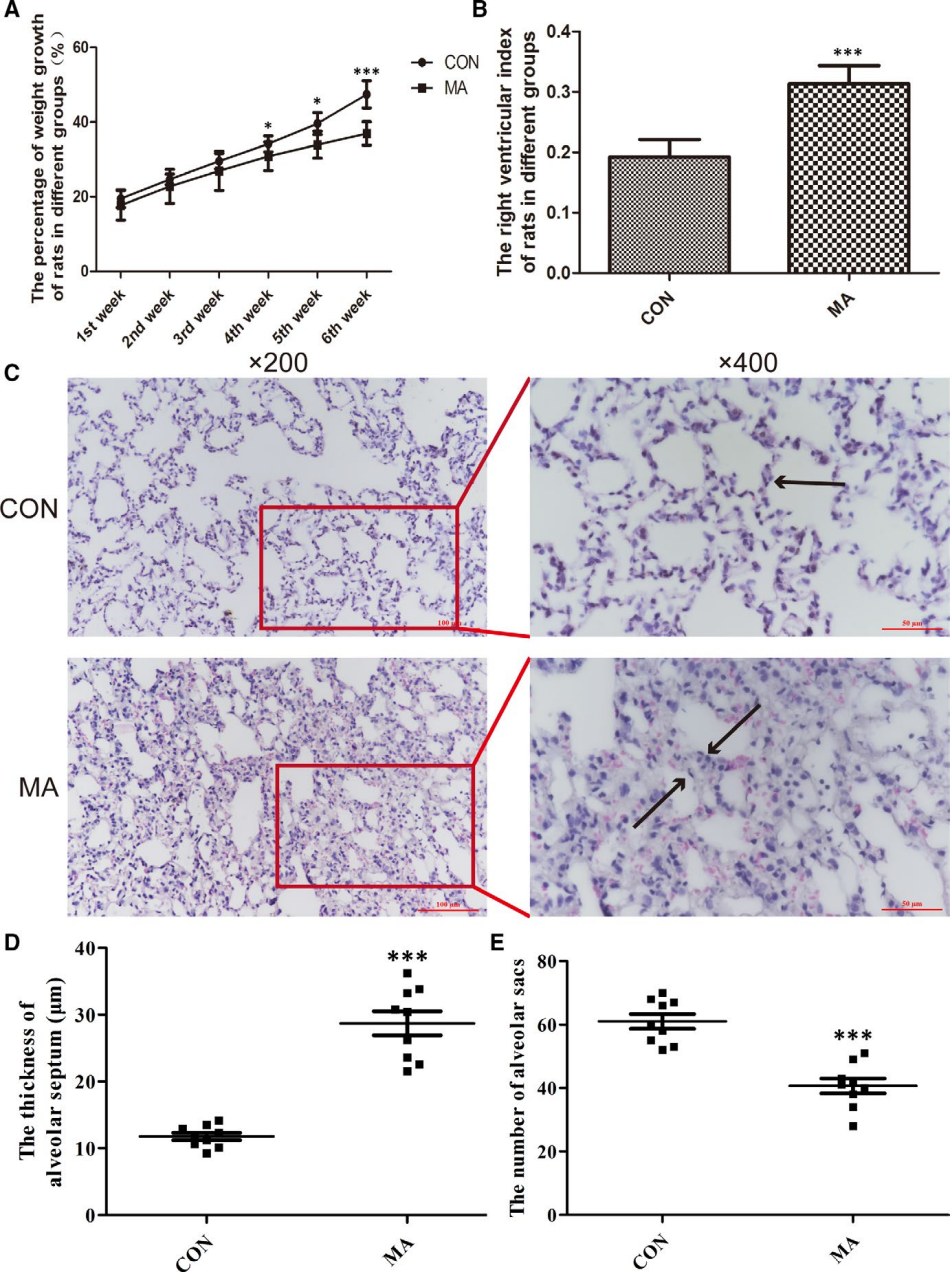
Chronic exposure to MA induced pulmonary injury in rats. A, The percentage of weight growth of rats in different groups. B, The right ventricular index of rats in different groups. C, MA induced chronic lung injury by H&E staining (Nikon, ECLIPSE TE2000‐U, Japan, ×200 and ×400). The black arrows represent the alveolar septum. D, The alveolar septum thickness (μm) in different groups (magnification, ×400). E, The number of alveolar sacs in different groups (magnification, ×200). The quantification of chronic lung injury was analysed in three fields of view randomly selected in each section. Data are shown as mean ± SD, n = 7, *P < .05, ***P < .001, vs CON; CON, control group; MA, methamphetamine group

For 6 weeks of administration with MA, the rat weight growth in MA group had been declined, especially from the 4th to 6th week (Figure 1A). At the end of the 6th week, the RVI was significantly increased in MA group, compared with control group (Figure 1B), which indicated that it was successful to establish the chronic toxicity model of MA.

The results from H&E staining suggested that chronic exposure to MA can cause more compact parenchyma of lungs (Figure 1C), including the thickened alveolar septum (Figure 1D) and reduction of the number of alveolar sacs (Figure 1E).

### 3.2 MA‐induced autophagy in lungs and alveolar epithelial cells

**FIGURE 2:**
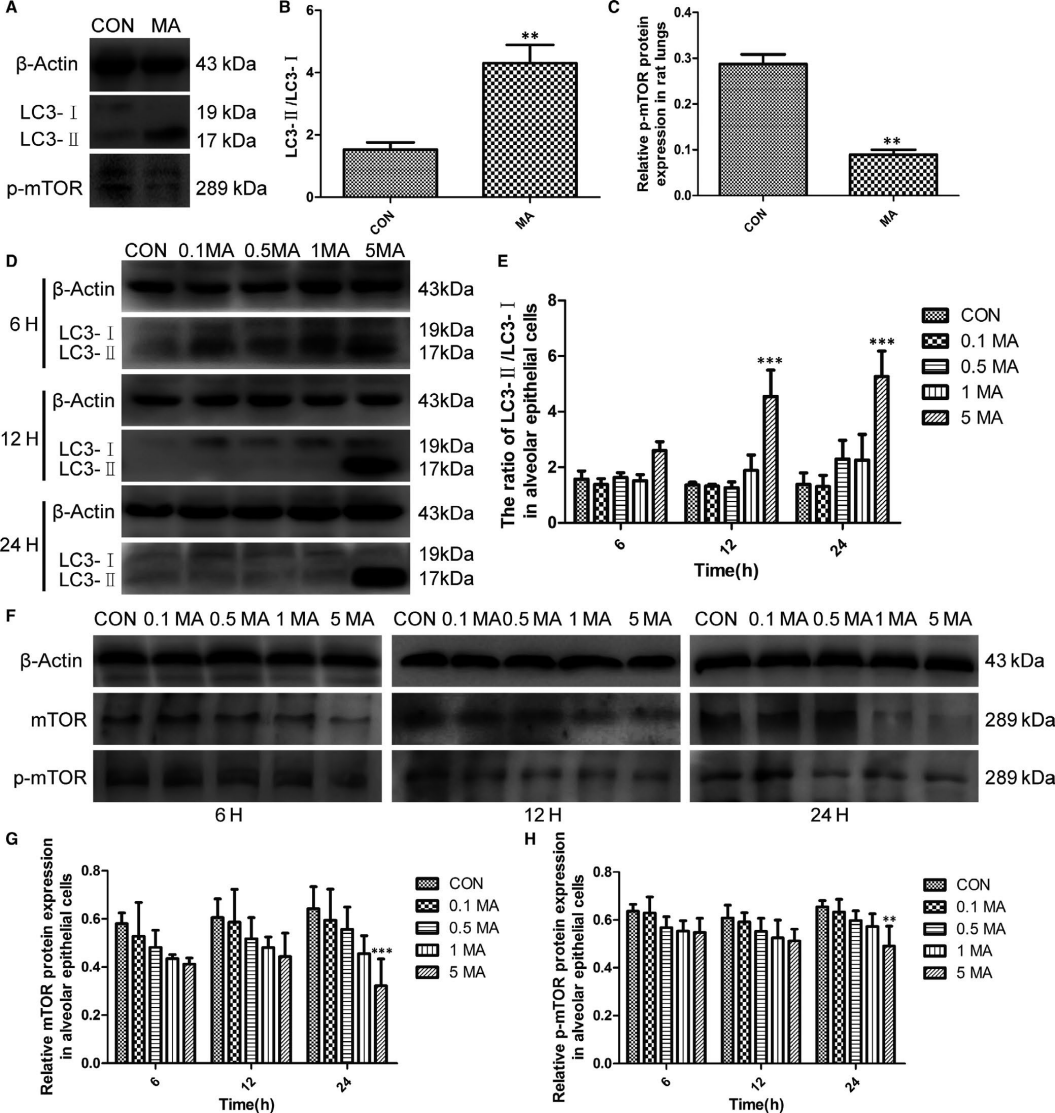
MA induced autophagy in lungs and alveolar epithelial cells. A, Western blot analysis of LC3 and p‐mTOR in lungs. B, The ratio of LC3‐II/LC3‐I protein in rat lungs. C, Relative p‐mTOR protein expression in rat lung tissue. Alveolar epithelial cells were treated with 0.1, 0.5, 1 and 5 mmol/L MA for 6, 12 and 24 h, respectively. D, The effect of MA on the expression of LC3 in alveolar epithelial cells. E, The ratio of LC3‐II/LC3‐I in alveolar epithelial cells. F, Western blot analysis of mTOR, and p‐mTOR in different groups. G, Relative mTOR protein expression in alveolar epithelial cells. H, Relative p‐mTOR protein expression in alveolar epithelial cells. Data were shown as means ± SD, **P < .01, ***P < .001 vs CON. CON, control group; 0.1 MA, 0.1 mmol/L MA group; 0.5 MA, 0.5 mmol/L MA group; 1 MA, 1 mmol/L MA group; 5 MA: 5 mmol/L MA group; MA, methamphetamine

LC3 is a component of the autophagosome and represents the occurrence of autophagy.^37^, ^38^ Once autophagy is activated, LC3‐I is converted to LC3‐II. LC3‐Ⅱ/LC3‐Ⅰ ratio is widely used as a marker of autophagy activation.^39^ In this study, it was found that compared with control group, LC3‐Ⅱ expression was significantly increased (Figure 2A), and the ratio of LC3‐Ⅱ/LC3‐Ⅰ was up‐regulated in MA group (Figure 2B). The phosphorylation of mTOR was inhibited by MA, and the expression of p‐mTOR was markedly reduced (Figure 2A,C), so that mTOR was inactivated to induce autophagy in lungs.

Western blot analysis showed that in alveolar epithelial cells, the protein levels of LC3‐Ⅱ were notably increased at 5 mmol/L MA, compared with control groups (Figure 2D). The ratio of LC3‐Ⅱ/LC3‐Ⅰ in 5 mmol/L MA group for 24 hours was much higher than other groups (Figure 2E). Therefore, LC3‐Ⅱ/LC3‐Ⅰ was dose‐dependently and time‐dependently increased by MA in alveolar epithelial cells, which is consistent with that in lungs of rats. The protein expressions of mTOR and p‐mTOR were remarkable decreased at 5 mmol/L MA for 24 hours, compared with control groups (Figure 2F‐H). The results indicated that exposure to MA can suppressed mTOR activation dose‐dependently and time‐dependently.

### 3.3 Protein‐protein interactions exist between IMP1 and mTOR

IMP1 is involved in many signalling pathways and cellular processes.^19^ Studies have shown that mTOR participates in the regulation of IGFs axis, that mTOR mRNA can bind to IMP1 by RNA immunoprecipitation and that mTOR protein can catalyse IMP1 phosphorylation at ser181 site.^30^, ^40^ Therefore, there may be a closer relationship between mTOR and IMP1.

**FIGURE 3:**
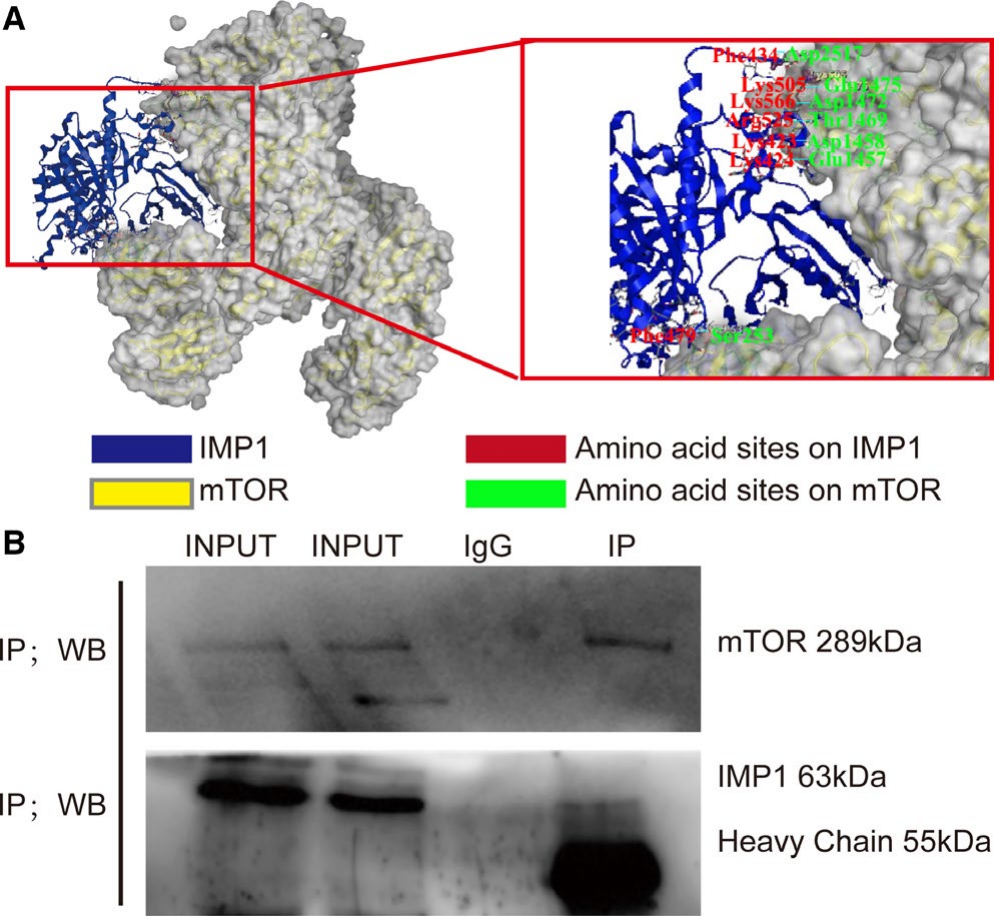
Protein‐protein interactions exist between IMP1 and mTOR. A, The results of bioinformatics prediction between IMP1 and mTOR. There are seven pairs of hydrogen bonds between IMP1 and mTOR, shown as amino acid sites on them. B, The results of co‐immunoprecipitation between IMP1 and mTOR. Samples were made by capture mTOR protein with agarose beads, and IMP1 protein was detected by Western blot analysis

MOE software was carried out to predict the molecular docking between IMP1 and mTOR. We obtained the protein structure of IMP1 and mTOR from Protein Data Bank (http://www.rcsb.org/) (Figure 3A). In the most stable conformation, it was synthetically evaluated that there are seven pairs of hydrogen bonds between the two proteins (Table 2). It was implied that there may be a link between IMP1 and mTOR. The results from co**‐**immunoprecipitation were that IMP1 was also dragged down after mTOR was pulled down by agarose (Figure 3B), which supported the bioinformatics prediction and indicated that there was protein‐protein interactions between IMP1 and mTOR.

**TABLE 2:** The binding sites between IMP1 and mTOR by bioinformatics prediction

mTOR	IMP1
Ser253	Phe479
Thr1469	Arg525
Glu1457	Lys424
Asp1458	Lys423
Asp1472	Lys566
Glu1475	Lys505
Asp2517	Phe434

### 3.4 MA caused the senescence in lungs

**FIGURE 4:**
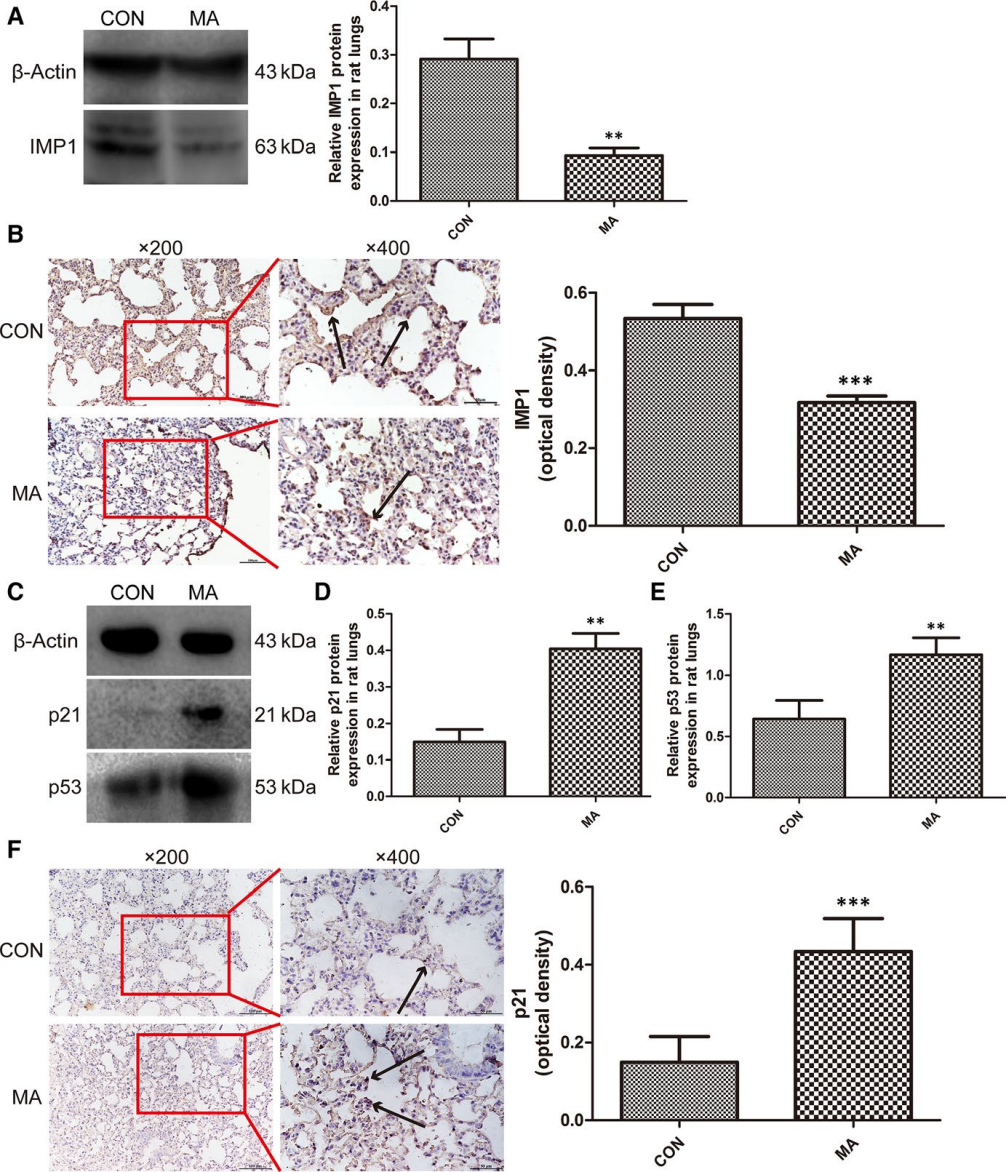
MA caused the senescence in lungs. A, Western blot analysis of IMP1 in lungs. B, IHC results of IMP1 expression in lungs (magnification, ×200, ×400). The brown represented the expression of IMP1 located on alveolar epithelium with the black arrows. C, Western blot analysis of p21 and p53 in lungs. D, Relative expression of p21 in lungs. E, Relative expression of p53 in lungs. F, IHC results of p21 expression in lungs (magnification, ×200, ×400). The black arrows represented the expression of p21. Data were shown as means ± SD. **P < .01, ***P < .001 vs CON; CON, control group; MA, methamphetamine group

The relative protein expression of IMP1 in the rat lungs was shown by Western blot analysis (Figure 4A). In MA group, the IMP1 expression was significantly declined compared with control group. The IHC results were also indicated that IMP1 was mainly located on the alveolar epithelium and that MA markedly reduced the expression of IMP1 in lungs (Figure 4B). The senescence is regulated through p53‐p21‐pRb pathway, so p53 and p21 can be used as the markers of senescence.^41^ The protein expressions of p21 and p53 in rat lungs in MA group were nearly twice as much as that in control group (Figure 4C‐E). IHC results showed that the expression of p21 in the MA group was much higher than that in control group (Figure 4F), which indicated that MA could regulate the senescence‐associated factors and lead to pulmonary senescence.

### 3.5 MA‐induced cellular senescence in human alveolar epithelial cells

Senescent cells can be identified by the expression of the above markers or SA‐β‐gal activity.^41^ When cellular senescence occurs, the cell cycle is arrested with the increasing of G0/G1 phase and the decreasing of S phase, which can be induced by p53 via p21/CDKN1A‐dependent inhibition of cyclin A/E‐CDK2.^42^

**FIGURE 5:**
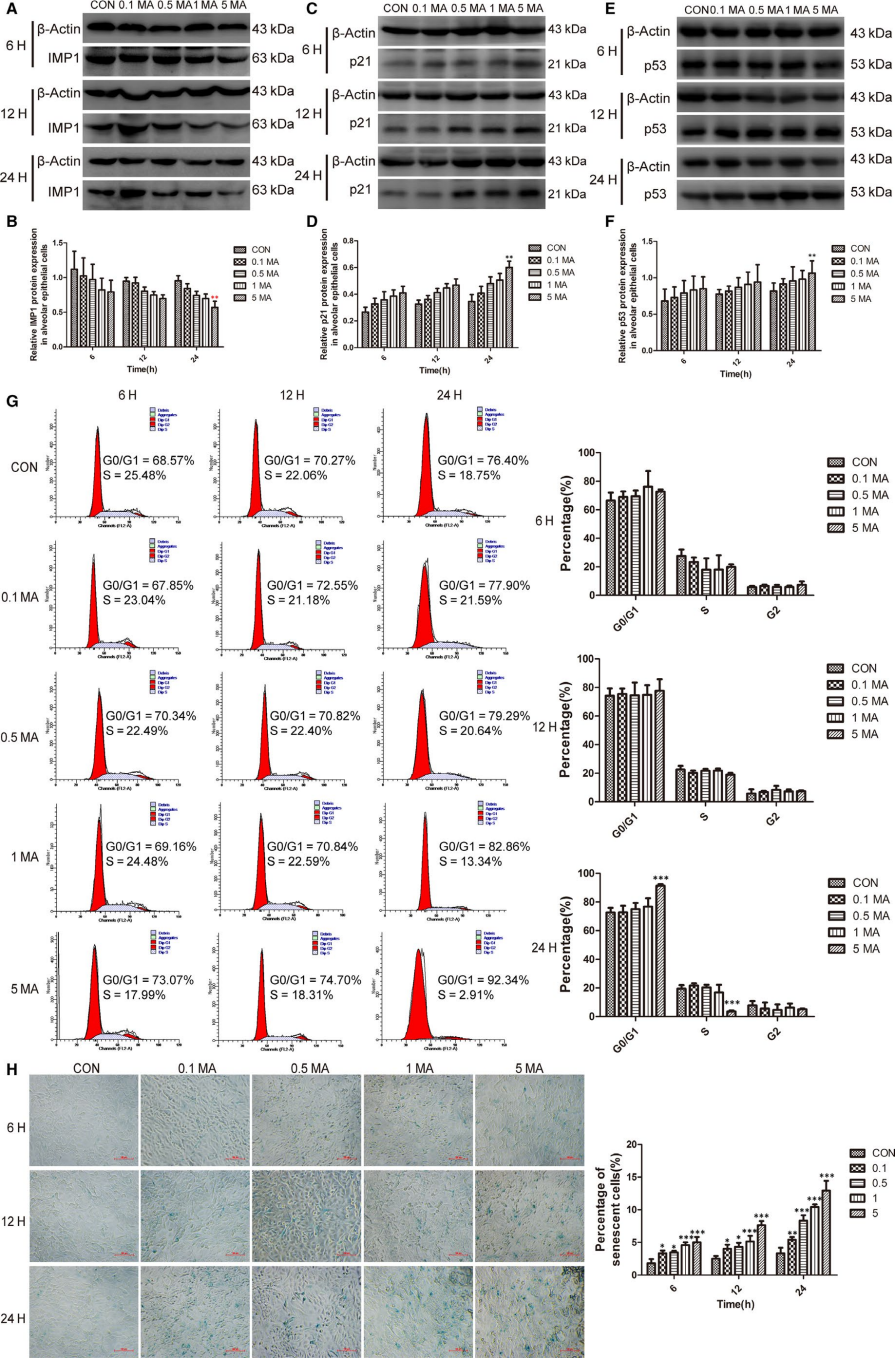
MA induced cellular senescence in alveolar epithelial cells. A‐B, Western blot analysis of IMP1 protein expression in alveolar epithelial cells in different groups. C‐D, Western blot analysis of p21 expression in alveolar epithelial cells in different groups. E‐F, Western blot analysis of p53 protein expression in alveolar epithelial cells in different groups. G, The effects of MA on cell cycle detection of alveolar epithelial cells by flow cytometry. H, The results and analysis of SA‐ß‐gal staining of alveolar epithelial cells (magnification, ×200). The blue‐stained cells were identified as senescent cells and were counted in three fields of view randomly selected in each section to determine the percentage of ß‐gal‐positive cells. Data were shown as means ± SD. *P *< .05*, **P < .01, ***P < .001 vs CON. CON, Control group; 0.1 MA, 0.1 mmol/L MA group; 0.5 MA, 0.5 mmol/L MA group; 1 MA, 1 mmol/L MA group; 5 MA: 5 mmol/L MA group; MA, methamphetamine

The relative protein expression of IMP1 in the alveolar epithelial cells was shown by Western blot analysis (Figure 5A). The protein expression of IMP1 was decreased significantly in cells at 5 mmol/L MA for 24 hours (Figure 5B). In alveolar epithelial cells, the protein expression of p21 was elevated markedly at 5 mmol/L MA for 24 hours in comparison with the control group (Figure 5C,D) and the protein expression of p53 was remarkably increased at 5 mmol/L MA for 24 hours, compared with control group (Figure 5E,F), which is identical with the results in lungs. It was suggested that the senescence‐associated p53‐p21 pathway was activated under the stimulation of MA with the prolongation of action time and the increase of drug concentration to induce the senescence.

The results from cell cycle detection showed that the G0/G1 phase was significantly raised but the S phase was markedly suppressed in 5 mmol/L MA for 24 hours (Figure 5G). The results hinted that MA made the cell cycle arrested at G0/G1 phase. It is further confirmed that long‐term and high‐dose administration of MA can induce cellular senescence.

Additionally, the SA‐ß‐gal staining results revealed that the percentage of senescent cells was augmented with the increasing of MA administration and actuation duration (Figure 5H). And there were statistically significance at nearly all the MA groups, compared with control group, which was implied that MA markedly increased the proportion of senescent cells to induce the senescence in alveolar epithelial cells.

### 3.6 The role of IMP1 in MA‐induced pulmonary senescence

**FIGURE 6:**
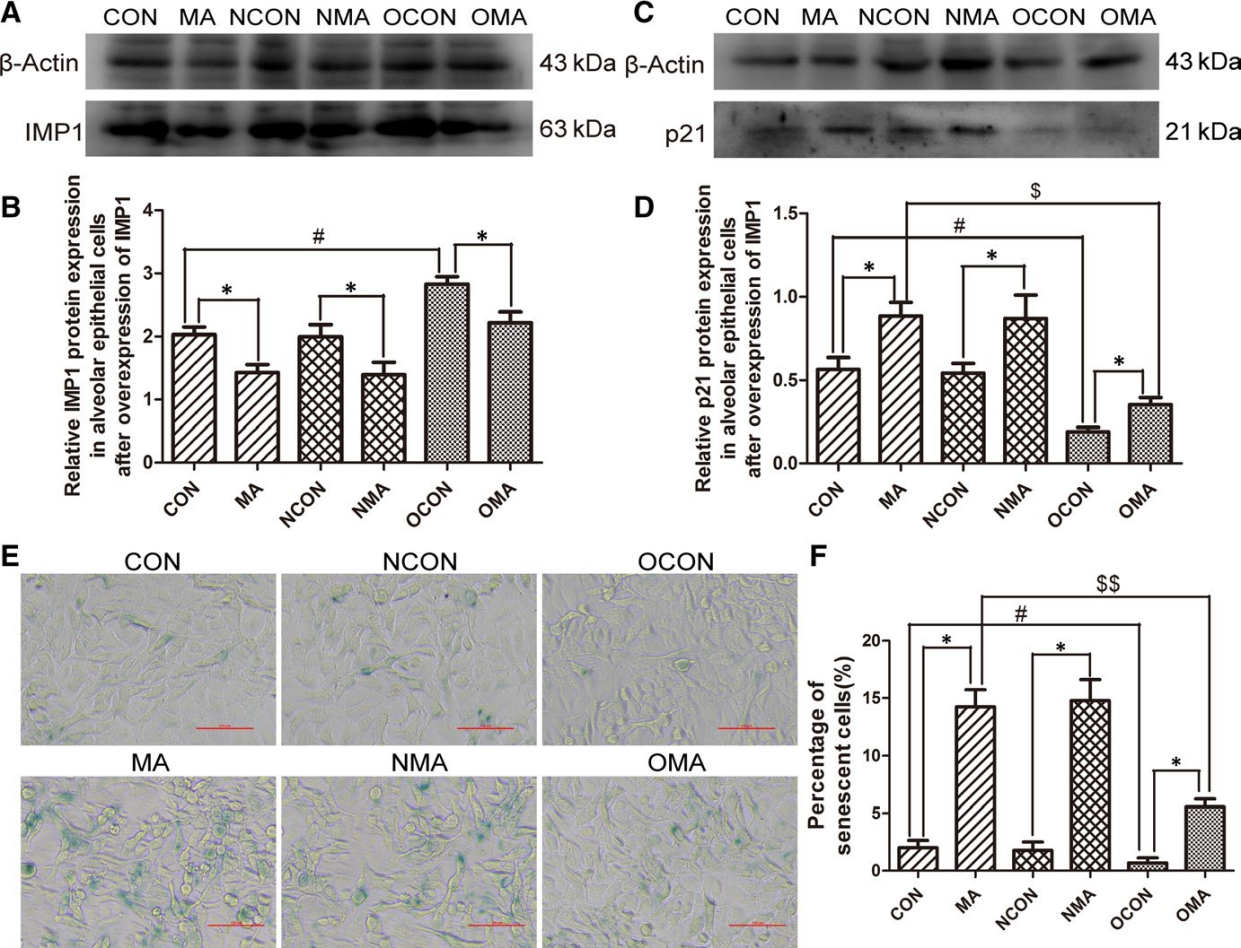
IMP1 played an important role in MA‐induced pulmonary senescence. IMP1 overexpression plasmids were transfected into alveolar epithelial cells, and then, the cells were treated with 5 mmol/L MA for 24 h. A, Western blot analysis of IMP1. B, Grey value analysis of IMP1. C, Western blot analysis of p21. D, Grey value analysis of p21. E, SA‐ß‐gal staining results of alveolar epithelial cells (magnification, ×200). F, The percentage of senescent cells. Data were shown as means ± SD. *P < .05 vs CON, NCON and OCON, respectively. ^#^ *P* < .05 vs CON. ^$^ *P* < .05, ^$$^ *P* < .01 vs MA. CON, control group; 0.1 MA, 0.1 mmol/L MA group; 0.5 MA, 0.5 mmol/L MA group; 1 MA, 1 mmol/L MA group; 5 MA: 5 mmol/L MA group; NCON, negative control group; NMA, negative control treated with MA; OCON, overexpression control group; OMA, overexpression group treated with MA; MA, methamphetamine

It was reported that in hepatocellular carcinoma, knockout of IMP1 can lead to a significant increase of p21, suggesting that IMP1 may influence the expression of p21.^34^ To investigate whether IMP1 can regulate p21, the IMP1 overexpression plasmids were transfected into the alveolar epithelial cells. After the incubation for 36 hours, 5 mmol/L MA was treated for 24 hours. Transfected efficiency was confirmed by Western blot analysis (Figure 6A,B). The results showed that in comparison with control group, IMP1 was significantly increased in over‐control group (^#^ *P* < .05, vs CON), which indicated that overexpression transfection is successful. Respectively compared with control group, negative control group or over‐control group, MA markedly reduced the expression of IMP1 (*P < .05 vs CON, NCON and OCON, respectively). And the results of negative control group and negative MA group indicated that blank plasmid had no effect on the experimental results.

Figure 6C,D showed that IMP1 overexpression notably suppressed the expression of p21 (^#^ *P* < .05, vs CON), and that compared with each control group, MA significantly increased the expression of p21 (*P < .05 vs CON, NCON and OCON, respectively) and that p21 level was markedly decreased in over‐MA group in the comparison with that in MA group (^$^ *P* < .05, vs MA).

The results of the SA‐ß‐gal staining were consistent with the change of p21 (Figure 6E). Compared with each control group, MA significantly augmented the expression of SA‐ß‐gal in alveolar epithelial cells (Figure 6F) (*P < .05 vs CON, NCON and OCON, respectively), which implied that MA induced cellular senescence. Contrasted with control group, SA‐ß‐gal in over‐control group was significantly declined (Figure 6F) (^#^ *P* < .05, vs CON), which suggested that overexpression of IMP1 can also down‐regulated the activity of SA‐ß‐gal by suppressing the expression of p21. With the comparison of MA group, the production of SA‐ß‐gal was significantly reduced in over‐MA group (Figure 6F) (^$$^ *P* < .01, vs MA), which demonstrated that overexpression of IMP1 can alleviate MA‐induced cellular senescence.

## 4 DISCUSSION

Although most studies of MA have been focused on the nervous system, about 78.3% deaths from methamphetamine toxicity are due to pulmonary diseases.^43^, ^44^ This study showed that long‐term exposure to MA led to more compact lungs, such as the thickening of alveolar walls and reduced number of alveolar sacs. MA promoted the conversion of LC3‐I to LC3‐II, inhibited the activation of mTOR to induce autophagy. Bioinformatics and co‐immunoprecipitation results presented the interplay between IMP1 and mTOR. MA down‐regulated mTOR and caused the lower IMP1 by the interactions between IMP1 and mTOR, which were resulted in the occurrence of cellular senescence by the up‐regulation of p21 and p53, the arrested cell cycle and the increased activity of SA‐ß‐gal, which represented. Overexpression of IMP1 reduced the levels of p21 and decreased SA‐ß‐gal to inhibit the senescence of alveolar epithelial cells. These results demonstrated that mTOR‐autophagy promotes pulmonary senescence through IMP1 in chronic toxicity of methamphetamine.

Autophagy is a highly conserved process for cell protection in various types of cellular stress (including nutritional deficiency, ROS, endoplasmic reticulum stress, bacterial infection).^40^ Our previous study found that MA could induce endoplasmic reticulum stress and inflammatory lesions in rat lung tissue.^45^ mTOR can inhibit autophagy, and phosphorylation is its active form, so that the down‐regulation of mTOR or inhibition of phosphorylation means the occurrence of autophagy.^46^, ^47^ Autophagosomes are produced by autophagy.^37^, ^38^ And LC3 is one component of autophagosomes. When autophagy is activated, LC3‐I is converted into LC3‐II. The increasing of LC3‐II/LC3‐I ratio is widely used as a marker of autophagy activation.^39^ In this study, LC3‐II was increased significantly, and the ratio of LC3‐II to LC3‐I was obviously elevated. mTOR activation was also significantly suppressed after stimulation with MA. It implied that MA promoted pulmonary autophagy by accelerating the transformation from LC3‐I to LC3‐II and reducing the expression levels of p‐mTOR in both dose‐dependently and time‐dependently way.

Autophagy disorder is a key cause of many diseases, including neurodegenerative diseases and some metabolic diseases.^48^ Some researchers pointed out that since autophagy can be divided into general autophagy and selective autophagy, their regulatory mechanisms are different and they have different effects.^49^ Selective autophagy inhibits senescence by GATA4, a key regulator of the senescence‐associated secretory phenotype and senescence, while general autophagy induces senescence by TASCC (TOR‐autophagy spatial coupling compartment).^49^ But how does autophagy induce the senescence? IMP1 takes a part in many cell life processes, including cell proliferation and cell death.^19^ Therefore, IMP1 is likely involved in cellular senescence. We predicted the protein‐protein binding of IMP1 and mTOR by molecular docking software and found that there were a great possibility of protein‐protein interactions between IMP1 and mTOR, which were confirmed by co‐immunoprecipitation. The interplay between mTOR and IMP1 may be the linkage between autophagy and senescence.

Furthermore, it was found that the expressions of p21 and p53 were significantly increased by MA. The results from cell cycle detection and SA‐ß‐gal staining showed that MA with 5 mmol/L for 24 hours notably blocked the cell cycle G0/G1 phase and augmented the expression of SA‐ß‐gal in alveolar epithelial cells. It was confirmed that MA could also induce cellular senescence in alveolar epithelial cells does‐dependently and time‐dependently. Taken together, it is demonstrated that MA could induce not only autophagy but also senescence by chronic action. In order to furtherly identify the role of IMP1 in MA‐induced senescence, the plasmids of IMP1 overexpression were transfected into alveolar epithelial cells. It was found that overexpressed IMP1 reduced the expression of p21 and decreased SA‐ß‐gal and it can also reduce the increase of p21 and SA‐ß‐gal activity caused by MA. Hence, IMP1 had a negative regulatory effect on p21 and prevented the cellular senescence induced by MA.

In conclusion, it was discovered in this study that long‐term exposure to MA can induce pulmonary autophagy and cellular senescence which may be mediated by the interplay between mTOR and IMP1. Therefore, mTOR‐autophagy promotes pulmonary senescence through IMP1 in chronic toxicity of methamphetamine.

## CONFLICT OF INTEREST

The authors declare no conflict of interest.

## AUTHOR CONTRIBUTIONS

**Mei‐Jia Zhu:** Conceptualization (equal); Data curation (equal); Formal analysis (equal); Software (equal); Validation (equal); Visualization (equal); Writing‐original draft (equal). **Bing‐Yang Liu:** Methodology (equal); Resources (equal). **Lin Shi:** Investigation (equal); Software (equal); Validation (equal). **Xin Wang:** Investigation (equal); Software (equal); Validation (equal). **Yun Wang:** Conceptualization (equal); Funding acquisition (equal); Methodology (equal); Project administration (equal); Resources (equal); Supervision (equal); Visualization (equal); Writing‐review & editing (equal).

## Data Availability

The data that support the findings of this study are available from the corresponding author upon reasonable request.
